# Low glutaminase and glycolysis correlate with a high transdifferentiation efficiency in mouse cortex

**DOI:** 10.1111/cpr.13422

**Published:** 2023-02-14

**Authors:** Yuan Li, Tingting Yang, Yingying Cheng, Jinfei Hou, Zhaoming Liu, Yu Zhao, Shiyu Chen, Zhaohui Qin, Chenchen Wang, Weining Song, Haofei Ge, Changpeng Li, Lining Liang, Lin Guo, Hao Sun, Lin‐Ping Wu, Hui Zheng

**Affiliations:** ^1^ Guangdong Provincial Key Laboratory of Stem Cell and Regenerative Medicine, GIBH‐CUHK Joint Research Laboratory on Stem Cell and Regenerative Medicine, Centre for Regenerative Medicine and Health Hong Kong Institute of Science & Innovation, Guangzhou Institutes of Biomedicine and Health, Chinese Academy of Sciences Guangzhou China; ^2^ University of Chinese Academy of Sciences Beijing China; ^3^ Department of Plastic Surgery The First Affiliated Hospital, Zhejiang University School of Medicine Hangzhou China; ^4^ Joint School of Life Sciences Guangzhou Medical University Guangzhou China; ^5^ Institutes for Stem Cell and Regeneration Chinese Academy of Sciences Beijing China

## Abstract

Both exogenous transcriptional factors and chemical‐defined medium can transdifferentiate astrocytes into functional neurons. However, the regional preference for such transdifferentiation has not been fully studied. A previously reported 5C medium was infused into the mouse cortex and striatum to determine the regional preference for transdifferentiation from astrocytes to neurons. The numbers of NeuN^+^GFAP^+^EdU^+^ cells (intermediates) and NeuN^+^EdU^+^ cells (end products) were determined by immunofluorescence to explore the regional preference of transdifferentiation. In addition, to optimize the delivery of the transdifferentiation medium, three key growth factors, insulin, bFGF and transferrin, were loaded onto chitosan nanoparticles, mixed with gelatin methacryloyl and tested in animals with motor cortex injury. A higher transdifferentiation efficiency was identified in the mouse cortex. Differences in cellular respiration and the balance between glutaminase (*Gls*) and glutamine synthetase were confirmed to be key regulators. In addition, the sustained drug release system induced transdifferentiation of cortex astrocytes both in vivo and in vitro, and partially facilitated the behaviour recovery of mice with motor cortex injury. We also applied this method in pigs and obtained consistent results. In summary, low *Gls* and glycolysis can be used to predict high transdifferentiation efficiency, which may be useful to identify better indications for the current transdifferentiation system. In addition, the current drug delivery system has the potential to treat diseases related to cortex injuries.

## Graphical abstract

More NeuN^+^GFAP^+^EdU^+^ cells (intermediates) and NeuN^+^EdU^+^ cells (end products) were induced in the mouse cortex than striatum after 5C medium infusion. Low glycolysis and Gls expression were important for the higher transdifferentiation efficiency in the cortex. Three key growth factors, insulin, bFGF and transferrin, were loaded onto chitosan nanoparticles and mixed with gelatin methacryloyl hydrogels, which induced the transdifferentiation of astrocytes and facilitated the behavioural recovery of mice with motor cortex injury.

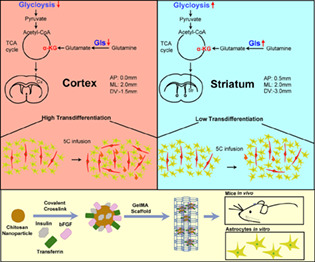

## 1 INTRODUCTION

Since Yamanaka reprogrammed mouse embryonic fibroblasts (MEFs) into induced pluripotent stem cells (iPSCs) in 2006, cell fate conversion has become a promising strategy of cell therapy.^1^ Most brain‐related diseases can be attributed to the death of neurons that lack the capability for regeneration. Thus, it is important to regenerate new neurons and reconstruct neural circuits in injured or degenerated brains. After Wernig et al. used three transcription factors, *Ascl1*, *Brn2* and *Myt1l*, to transdifferentiate MEFs into functional neurons in vitro,^2^ scientists established a variety of methods to convert different types of cells into functional neurons. In addition to MEFs, astrocytes and NG2 glia cells were transdifferentiated into neurons in vitro and/or in vivo.^3^, ^4^ In addition to cell‐specific transcription factors, scientists found that decreasing genomic DNA methylation by upregulating the DNA dioxygenase TET3 can convert MEFs into neurons.^5^ Recently, transdifferentiation from astrocytes to neurons has been considered as a potential route to treat different types of neuronal diseases.^6^

However, the genomic integration of viruses that deliver exogenous transcription factors into the mouse brain raises many concerns about the safety of the clinical application of this strategy. In contrast, small‐molecule compound‐induced cell fate conversion is relatively easier to standardize and optimize. In 2015, Deng's laboratory established a new cocktail of small‐molecule compounds to convert MEFs into neurons.^7^ Additionally, Chen's team established a combination of small‐molecule compounds to successfully transdifferentiate astrocytes into neurons.^8^ Our laboratory has also reported that transdifferentiation can be achieved by a chemically defined medium (5C medium; F12 supplanted with N2, bFGF, leukaemia inhibitory factor, vitamin C and 2‐mercaptoethanol) in the mouse brain.^9^

Recently, several studies have debated the source of generated neurons.^10^ Chen's laboratory used additional experiments to prove that the generated neurons were directly converted from astrocytes in situ.^11^ Although controversies still exist in the field, we believe that the transdifferentiation from astrocytes to neurons has a promising future in treating neuronal diseases.

Although Deng's laboratory showed that neurons newly generated in situ by small‐molecule compounds resembled the characteristics of region‐specific subtypes,^12^ whether transdifferentiation has a regional preference in the mouse brain has not been fully determined. Astrocytes in different regions of the mouse brain differ greatly,^13^ which may result in their different responses to transdifferentiation methods. Therefore, it is reasonable and necessary to determine the regional preference of the current transdifferentiation methods.

Cellular metabolism is crucial in neuronal transdifferentiation and differs greatly in astrocytes from different regions.^13^, ^14^ In addition, the metabolic balance of glutamine affects the tricarboxylic acid (TCA) cycle and oxidative phosphorylation (OXPHOS).^15^ Glutaminase (GLS) catalyses the catabolism of glutamine to glutamate, which is the rate‐limiting and the first step of glutaminolysis.^16^, ^17^ Glutamine synthetase (GLUL) converts glutamate and ammonia to glutamine. The relative expression of *Gls* and *Glul* influences cellular metabolism and participates in a variety of pathological processes.^18^, ^19^, ^20^ The potential contributions of GLS and GLUL to transdifferentiation in different regions were determined in the current studies.

## 2 MATERIALS AND METHODS

### 2.1 Composition of the current medium

MEF medium included high‐glucose DMEM, 10% foetal bovine serum (10%), 1% non‐essential amino acids and 1% GlutaMax. 5C medium included DMEM/F12, 1% N_2_ supplement, 20 ng/mL bFGF, 1000 unit/mL leukaemia inhibitory factor, 50 μg/mL vitamin C and 55 μM 2‐mercaptoethanol. The 7C medium was 5C medium supplemented with 10 μM BPTES (bis‐2‐(5‐phenylacetamido‐1,3,4‐thiadiazol‐2‐yl)ethyl sulphide) and 5 mM 2‐deoxy‐d‐glucose (2‐DG). The 3C medium included 10 mg/L insulin, 55 μg/mL transferrin and 55 μg/mL bFGF. All materials used in the current studies were listed in Table S1.

Primary astrocytes were generated from mice as previously described.^21^ Astrocytes were cultured in 5C medium for 12 days and the percentage of TuJ^+^ cells was determined to quantify the transdifferentiation. Plat‐E cells were cultured in MEF medium to produce *Gls* and *shHif1α*‐encoding virus with pMXs‐based retroviral vectors.^22^

### 2.2 Animal studies

All procedures related to animal studies were performed in accordance with NIH Publication No. 80‐23 and were approved by the IRB in Guangzhou Institutes of Biomedicine and Health (No. IACUC‐2016018 for mouse, No. IACUC‐2020015 for pig). C57BL/6 mice were purchased from Beijing Vital River Laboratory Animal Technology. Pigs were purchased from Laboratory Animal Center, Southern Medical University.

### 2.3 Preparation and implantation of loaded chitosan nanoparticles with gelatin methacryloyl

The drug‐loaded nanoparticles were prepared via the ionic gelation method. Briefly, the chitosan nanoparticles (CNPs) were dissolved in a 1% acetic acid solution to make a solution at a concentration of 0.1%. Then, the pH of the solution was adjusted to 5.0–6.0, and moderate insulin, transferrin and bFGF were added to be absolutely dissolved. Sodium triphosphate solution (100 μL, 0.08%, pH 7–8) was added to 4 mL of the solution above under slow stirring. After stirring for at least 1 h, the nanoparticle solution was prepared, and gelatin methacryloyl (GelMA) was dissolved to make the hydrogel with loaded CNPs of 4% concentration. Vehicle and loaded CNPs were mixed with GelMA to form vehicle and loaded CNPG (CNP with GelMA), respectively. In addition, the 0.1% photoinitiator LAP was added. The crosslinking of GelMA with nanoparticles was processed under blue light (wavelength at 405 nm) and illuminated for 2 min. The sizes and zeta‐potential of particles were measured by the laser particle analyzer (Mastersizer 3000, England).

Eight‐ to ten‐week‐old C57 male mice were divided into three groups: a group implanted loaded CNPG (12 mice), a group implanted vehicle CNPG (12 mice) and a group received injury only (control, 12 mice). Mice were as described above. A bone window of 1.5 mm × 1.5 mm was drilled with a skull drill and a block of cortical tissue (1.5 mm × 1.5 mm × 1.5 mm) was removed mechanically by an excavator spoon to create a lesion cavity in the right motor cortex. Thrombin‐soaked gelfoam was used to control haemostasis. The loaded or vehicle CNPG was injected into the lesion cavity, and then photocrosslinked by blue light (405 nm, 2 min). The bone window was then covered with dental cement and the scalp was sutured. The mice were given antibiotics (ampicillin, 50–100 mg/kg) at the end of the implantation. EdU (5 mg/kg) dissolved in 0.9% sterile saline was injected intraperitoneally. EdU was administered to animals once a day for seven consecutive days.

Two‐month‐old Bama mini‐pigs were used for implantation. The intersection of the line between the eyes and the midline of the skull was considered as the origin of the location. A block of cortical tissue (7 mm × 7 mm × 7 mm) of motor cortex (AP: −18 to −25 mm, ML: 20–27 mm, DV: −7 mm) was removed to create a lesion cavity. The procedures of the transplanted L‐CNPG were similar to those in mice.

### 2.4 Infusion of 5C and 7C medium into mouse brain

For 5C infusion, 100 μL 5C medium (DMEM/F12 was 1×, other components were 50×) with 8 μg/mL EdU were loaded into the osmotic minipumps to obtain a constant release at the rate of 0.25 μL/h for 14 days (Alzet 1002; Cupertino, CA). In the control group, the same amount of F12 medium with EdU (8 μg/mL) was used. For 7C infusion, a total volume of 100 μL 7C medium (5C medium supplemented with 2‐DG and BPTES, DMEM/F12 was still 1×, but other components were 50×) with 8 μg/mL EdU were loaded into the osmotic minipumps to obtain a constant release at the rate of 0.25 μL/h for 14 days (Alzet 1002; Cupertino, CA). In the control group, the same amount of F12 medium with EdU (8 μg/mL) was infused into the mouse brains.

Eight‐week‐old C57BL/6 male mice were anaesthetized by intraperitoneal injection with ketamine (90 mg/kg) and xylazine (10 mg/kg) and then placed in a stereotactic device. The cannula was inserted into the cortex (AP: 0.0 mm, MP: 2.0 mm, DV: −1.5 mm) or striatum (AP: 0.5 mm, MP: 2.0 mm, DV: −3.0 mm) and the pump subcutaneously on the back. Animals were given antibiotics (ampicillin, 50–100 mg/kg) at the end of the implantation and placed in a clean warm cage on a heating pad until mobile. A total volume of 1 μL of rAAV‐CMV‐EGFP‐WPRE‐pA (1 × 10^13^ GC/mL, BrainVTA, China) was injected into the cortex (AP: 0.0 mm, MP: 2.0 mm, DV: −1.5 mm) through an infusion cannula at a flow rate of 40 nL/min.

### 2.5 Data and material availability

A previously reported dataset (GSE184933) with scRNA‐seq data from different brain regions was used in the manuscript.^23^ All material requirements could be addressed to Hui Zheng (zheng_hui@gibh.ac.cn).

### 2.6 Statistical methods

All statistic information in the current studies was listed in Table S2 and Data S1. In addition to the abovementioned methods, all other methods are listed in the Data S1.

## 3 RESULTS

### 3.1 5C medium induces higher transdifferentiation in the cortex

**FIGURE 1:**
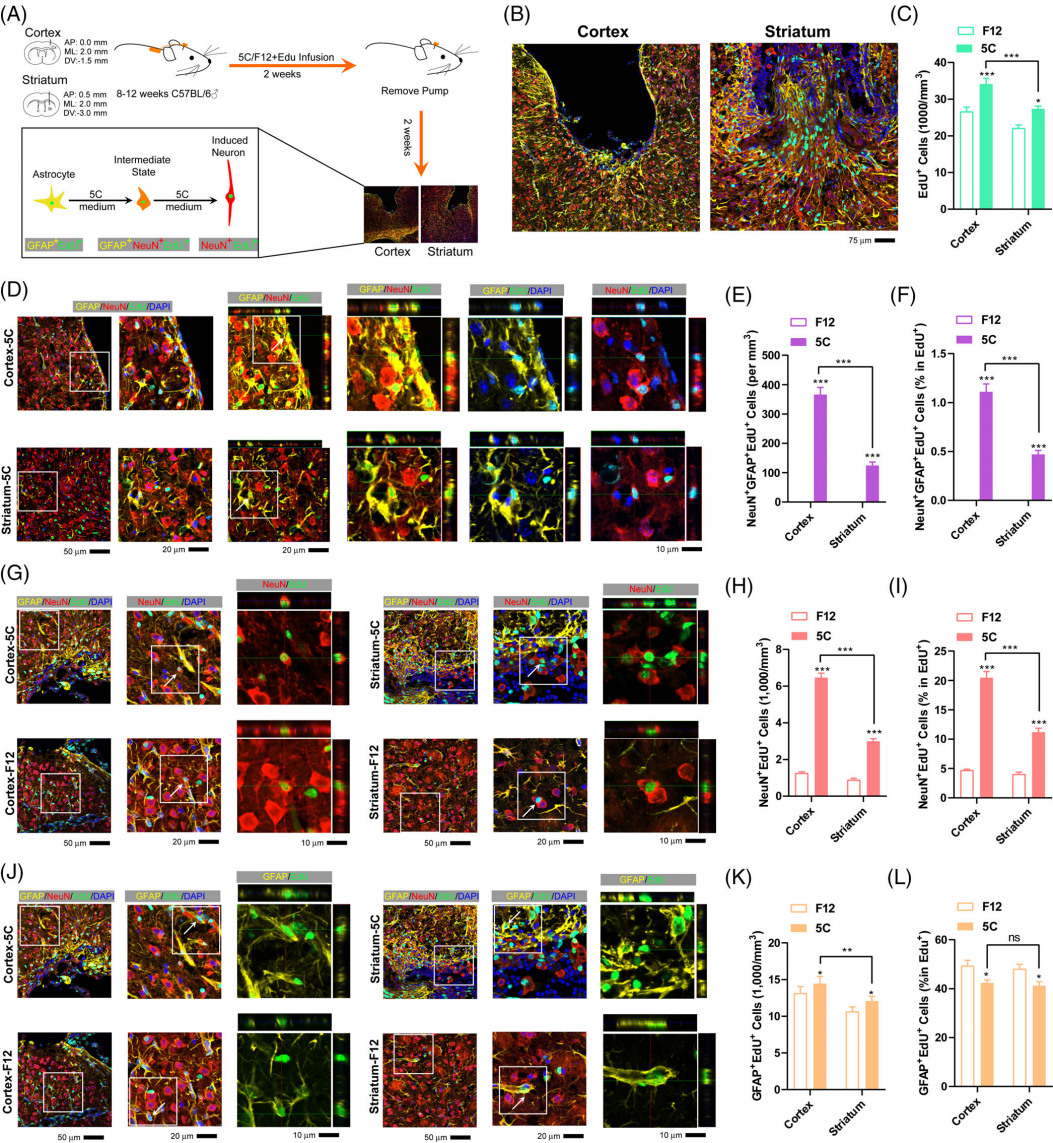
The 5C medium induces higher transdifferentiation in the cortex. (A) Experimental design for implantation of osmotic minipumps into the brain, EdU was delivered simultaneously with 5C medium. (B) Immunofluorescence around the injury sites at 4 weeks after 5C infusion in the cortex and striatum. (C) Quantification of total EdU^+^ cells in the peri‐injury core areas of the control (F12) and 5C group at 4 weeks after 5C infusion in the cortex and striatum. (D–F) Immunofluorescence for NeuN^+^GFAP^+^EdU^+^ cells in the cortex and striatum at 4 weeks (D). The amounts of these cells (E) and their percentage in EdU^+^ cells (F) were determined. (G–L) Analyses for NeuN^+^EdU^+^ cells (G–I) and GFAP^+^EdU^+^ cells (J–L) were performed similarly as in (D–F). Each group included at least six mice and at least seven frozen sections from each mouse were analyzed (*n* ≥ 42). Error bars represented standard errors. Two‐way ANOVA was used. Additional statistic information could be found in Table S2.

To determine the regional preference of the transdifferentiation induced by 5C medium, 5C medium (50×) was infused into two regions of the mouse brain, the motor cortex and striatum. The minipumps were removed 2 weeks after the surgery, and immunofluorescence was performed to quantify the transdifferentiation after an additional 2‐week recovery (Figure 1A).

Constitutive frozen sections around the injury sites were analyzed with immunofluorescence. Significantly more EdU^+^ cells were identified in mice infused with 5C medium (5C group) than in those infused with F12 medium (F12 group) (Figure 1B,C), suggesting that the infusion of 5C medium increased cell proliferation and might support subsequent transdifferentiation.^24^

NeuN and GFAP are widely reported and used as markers for mature neurons and astrocytes, respectively.^25^ Although there are nearly no NeuN^+^GFAP^+^ cells in the mouse brain under normal physical conditions, these double‐positive cells can be found during transdifferentiation from astrocytes to neurons both in vitro and in vivo.^9^, ^24^ NeuN^+^GFAP^+^EdU^+^ cells were seldom identified in the F12 group, but were much more abundant in the 5C group (Figure 1D,E).The percentage of NeuN^+^GFAP^+^EdU^+^ cells in EdU^+^ cells was also calculated and proved to be higher in the 5C group (Figure 1F). Therefore, the ability of 5C medium to induce transdifferentiation is higher in the cortex than in the striatum.

Since 5C medium‐induced transdifferentiation occurs from astrocytes to neurons, the starting material (GFAP^+^EdU^+^ cells) and the ending product (NeuN^+^EdU^+^ cells) of the transdifferentiation were also analysed. Significantly larger numbers of NeuN^+^EdU^+^ cells were observed in mice infused with 5C medium (Figure 1G–I). In addition, there were slightly fewer GFAP^+^EdU^+^ cells (percentage but not absolute number) in the 5C group than in the F12 group (Figure 1J–L). The reciprocal changes of these two types of cells at least partially confirmed the transdifferentiation from astrocytes to neurons in the cortex and striatum. The increase in the absolute number of GFAP^+^EdU^+^ cells in the 5C group was consistent with the previously reported ability of 5C medium to promote cell proliferation (Figure 1K).^24^ The decrease in the percentage of GFAP^+^EdU^+^ cells in EdU^+^ cells was consistent with the ability of 5C medium to convert GFAP^+^ cells to NeuN^+^ cells (Figure 1L).

### 3.2 5C medium induces direct transdifferentiation around the injury site

**FIGURE 2:**
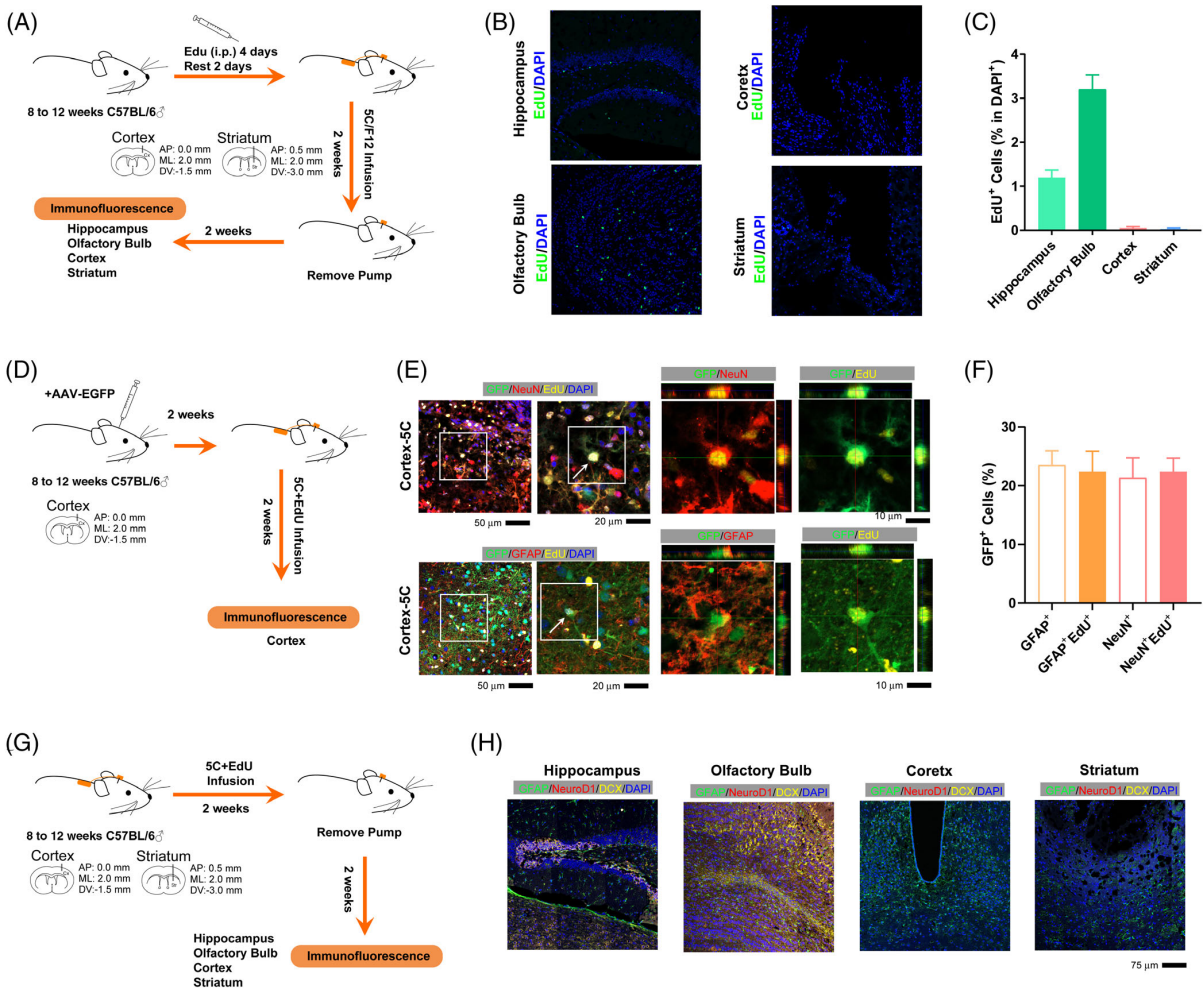
The 5C medium induces direct transdifferentiation around the injury site. (A) Experimental design for intraperitoneal injection of EdU and implantation of osmotic minipump into the brain. (B) Immunofluorescence for EdU (green) in the hippocampus, cortex, olfactory bulb and striatum. (C) Quantification of the percentage of EdU^+^ cells in DAPI^+^ cells in the hippocampus, cortex, olfactory bulb and striatum. (D) Experimental design for labelling with GFP‐encoding adenovirus before the 5C medium infusion. (E,F) Immunofluorescence and quantification for the abilities of GFP‐encoding adenovirus to label different types of cells, GFAP^+^, GFAP^+^EdU^+^, NeuN^+^ and NeuN^+^EdU^+^ cells. (G) Experimental design for staining against NeuroD1 and DCX around the injury site 2 weeks after minipump removing. (G) Immunofluorescence for GFAP (green), NeuroD1 (red) and DCX (yellow) in the hippocampus, cortex, olfactory bulb and striatum. Each group included at least three mice and at least four frozen sections from each mouse were analyzed (*n* ≥ 12). Error bars represented standard errors. Additional statistic information could be found in Table S2.

Several experiments were performed to eliminate the possibility that the NeuN^+^GFAP^+^ cells were differentiated from NSCs in the context of injury. In the first experiment, intraperitoneal injection of EdU was used to label the proliferating cells before the implantation of the osmotic minipump (Figure 2A). EdU injection successfully labelled NSCs in the hippocampus and olfactory bulb. However, few or even no EdU^+^ cells were identified around the injury sites (Figure 2B,C), suggesting that the EdU^+^ cells identified in Figure 1 were not from pre‐existing and proliferating cells or NSCs.

In the second experiment, a GFP‐encoding adenovirus was used to label the cells around the injury site immediately before the 5C medium infusion (Figure 2D). As indicated in Figure 2E,F, the GFP‐encoding adenovirus labelled GFAP^+^ and NeuN^+^ cells at efficiencies of ~20%, which was close to its abilities to label ~20% GFAP^+^EdU^+^ and NeuN^+^EdU^+^ cells. Thus, the majority of the proliferating and transdifferentiation‐related cells (EdU^+^ cells) were already located around the injury before the implantation of the minipump.

In the third experiment, we stained against NeuroD1 and Doublecortin (DCX) around the injury site 2 weeks after minipump removing. Low or even absent expression of NeuroD1 and DCX was detected (Figure 2G,H). Thus, the NeuN^+^GFAP^+^EdU^+^ cells were not from the quiescent NSCs around the injury site.

### 3.3 Low glycolysis contributes to the higher transdifferentiation rate in cortex

**FIGURE 3:**
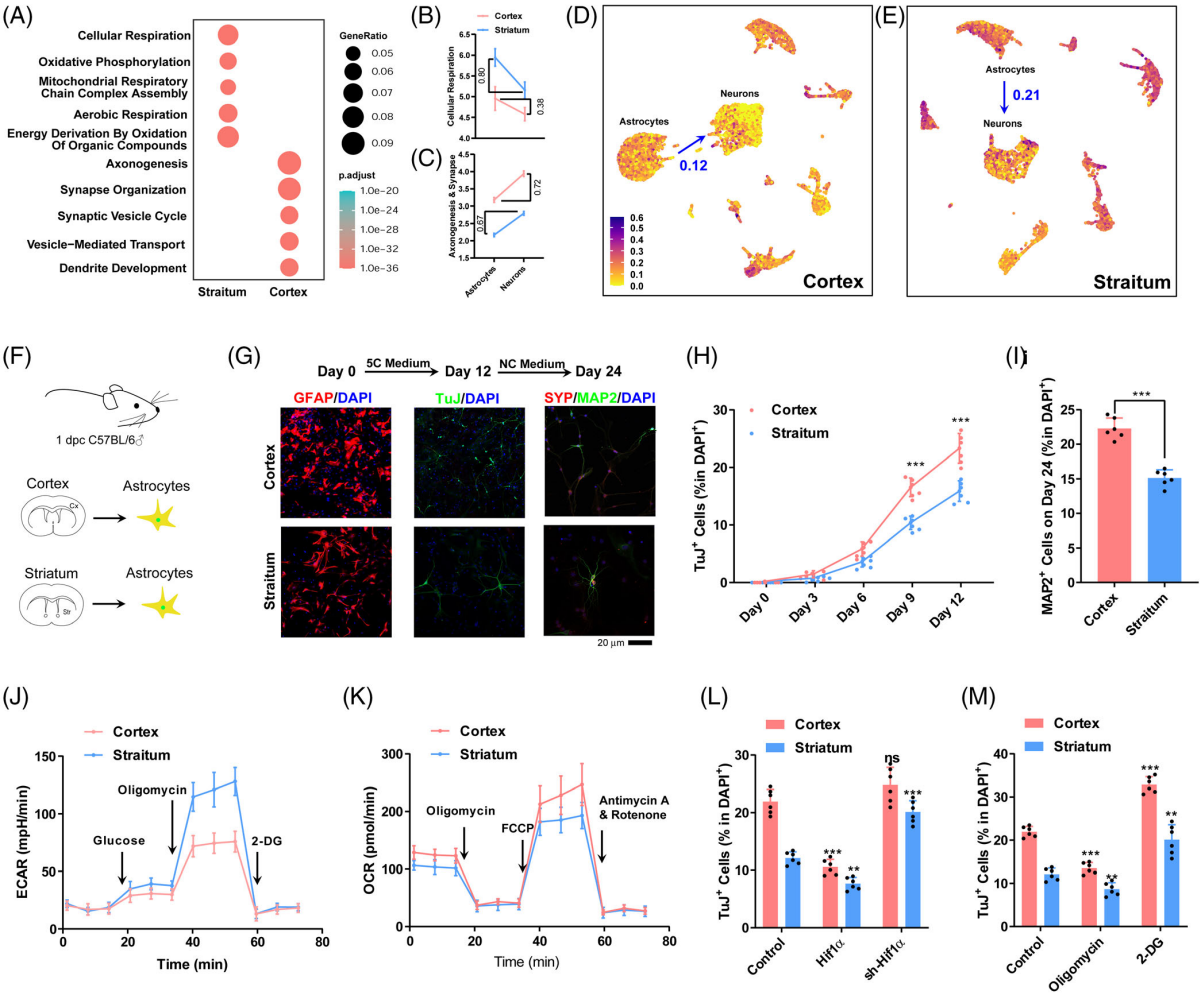
Low glycolysis contributes to the higher transdifferentiation rate in cortex. (A) Gene Ontology studies of differentially expressed genes between cortical and striatal astrocytes. (B,C) Expression changes of genes related to cellular respiration (B) and axonogenesis and synapse (C) during transdifferentiation in the cortex and striatum. (D,E) Expression of genes related to cellular respiration in the cortex (D) and striatum (E) at single cell level. (F) Schematic overview of transdifferentiation in vitro. (G) Representative immunofluorescence images show that astrocytes can be converted into neurons by 5C medium and form mature neurons after incubation with NC medium for additional 12 days. (H,I) The percentage of TuJ^+^ cells and MAP2^+^ cells in DAPI^+^ cells. (J,K) Energy metabolism of primary astrocytes was analysed with a Seahorse instrument. (L,M) Efficiency of astrocyte transdifferentiation in the cortex and striatum after OXPHOS changes controlled by *Hif1α* gene up/downregulation and drugs (2‐DG/oligomycin). Experiments were repeated for at least six times (*n* ≥ 6) except scRNA‐seq from previous report. Error bars represented standard deviations. One‐way ANOVA and two‐way ANOVA were used for L,M and H, respectively, Student two‐tailed *t*‐test was used for I. Additional statistic information could be found in Table S2.

We then analyzed a previously reported dataset with scRNA‐seq data from different brain regions (GSE184933).^23^ Gene Ontology (GO) studies suggested that 835 genes with higher expression in both neurons and astrocytes in the cortex were enriched with terms related to axonogenesis and synapse organization, while 761 genes with lower expression were enriched with terms related to cellular respiration and OXPHOS (Figure 3A).

The changes in these genes during transdifferentiation were predicted by comparing their expression in neurons and astrocytes. Genes related to cellular respiration and OXPHOS should experience significantly larger expression changes during transdifferentiation in the striatum than in the cortex (Figure 3B,C), which might be an explanation for the higher transdifferentiation rate in the cortex. When analyzing the genes related to cellular respiration at single cell level, the differences between astrocytes and neurons in the cortex were smaller than those in the striatum (Figure 3D,E).

To confirm the hypothesis mentioned above, we isolated primary astrocytes from different regions of the mouse brain (Figure 3F). As indicated in Figure 3G,I, primary astrocytes from the cortex had higher transdifferentiation efficiency than those from the striatum. The metabolic characteristics of these primary astrocytes were determined with the Seahorse instrument (Figure 3J,K). Primary astrocytes from the cortex had a lower level of glycolysis and a lower level of OXPHOS than those from the striatum (Figure 3J,K). Exogenous expression of *Hif1α* promoted glycolysis and impaired OXPHOS,^22^ which subsequently inhibited the transdifferentiation of the two types of primary astrocytes. Suppressing *Hif1α* expression with sh‐RNA led to the opposite results (Figure 3L). In addition, 2‐DG and oligomycin were used to modulate OXPHOS and consistent results were obtained (Figure 3M). Therefore, the lower glycolysis and higher OXPHOS in primary astrocytes from the cortex contributed to its higher transdifferentiation rate.

### 3.4 GLS inhibits transdifferentiation by promoting glycolysis

**FIGURE 4:**
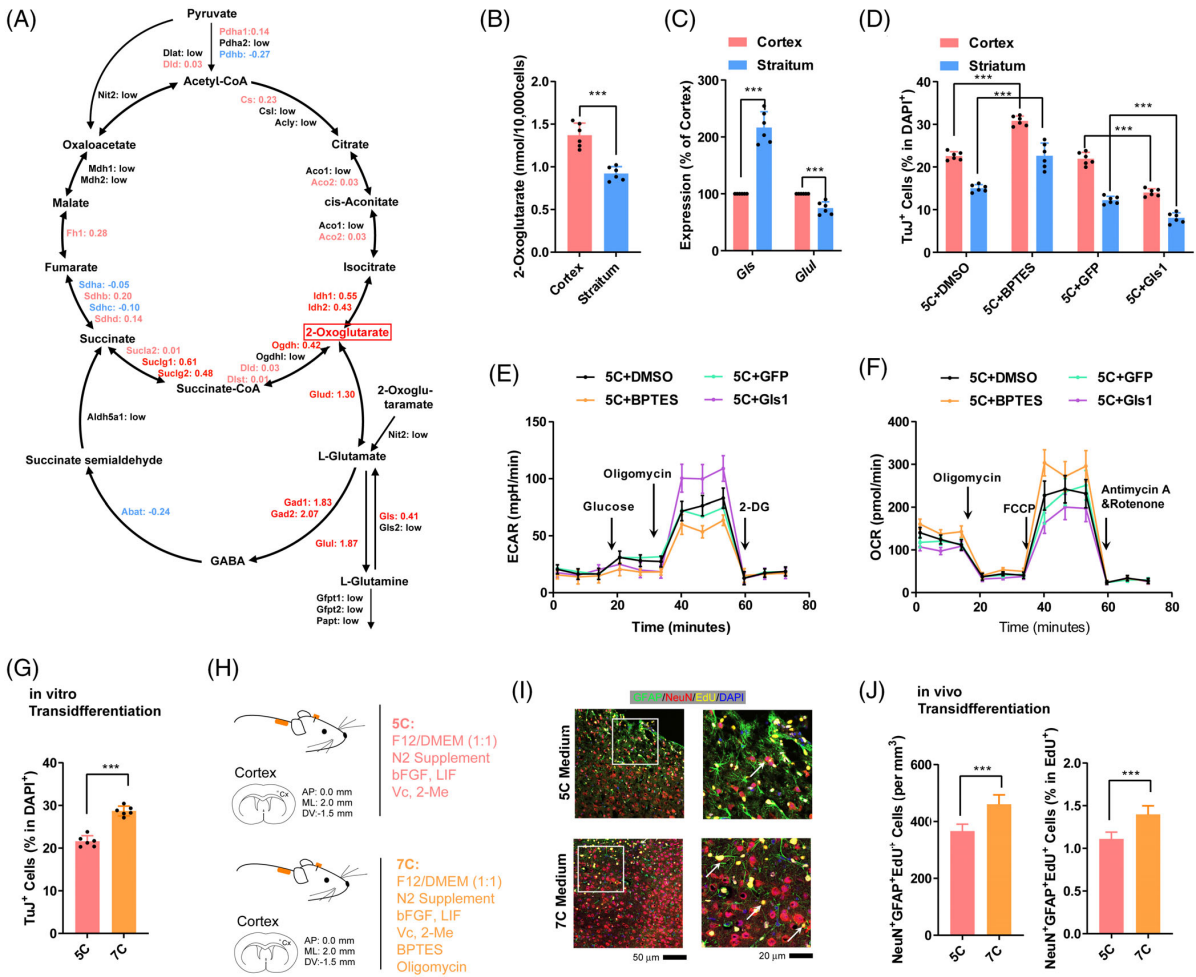
GLS inhibits transdifferentiation by promoting glycolysis. (A) Expression changes of genes in the cellular respiration‐related pathways were compared during transdifferentiation in the cortex and striatum. Red gene should experience larger changes during transdifferentiation in striatum. Blue genes functioned just oppositely. Black genes had low expression. (B) The intracellular concentration of 2‐oxoglutarate in astrocytes from the cortex and striatum. (C) Expression of *Gls1* and *Glul* genes in cortical and striatal astrocytes. (D) Transdifferentiation efficiencies of cortical and striatal astrocytes after *Gls1* overexpression or GLS1 suppression with BPTES. (E,F) Energy metabolism was analyzed in cortical astrocytes after *Gls1* overexpression or GLS1 suppression with BPTES with a Seahorse instrument. (G) Transdifferentiation efficiency of cortical astrocytes by 5C and 7C medium. (H–J) Schematic overview of 7C medium‐induced transdifferentiation in vivo (H). Representative immunofluorescence images of NeuN^+^GFAP^+^EdU^+^ cells were provided in (I) and the results were summarized in (J). Experiments were repeated for at least six times (*n* ≥ 6). Each group included at least six mice and at least seven frozen sections from each mouse (*n* ≥ 42) (I,J). Error bars represented standard deviations except standard errors in (J). Student two‐tailed *t*‐test was used except two‐way ANOVA were used for (D). Additional statistic information could be found in Table S2.

The important roles played by the balance between OXPHOS and glycolysis during transdifferentiation are consistent with a previous report.^14^ We then tried to identify key factors in pathways related to cellular respiration. As indicated in Figure 4A, red gene should experience larger changes during transdifferentiation in striatum and might be the reason for higher transdifferentiation in cortex. Blue genes might function just oppositely, while black genes had low expression. We found that several enzymes, including *Aco2*, *Idh1*, *Ogdh*, *Gls* and *Glul* which influence the intracellular level of 2‐oxoglutarate, in red genes (Figure 4A), suggesting that 2‐oxoglutarate might be a critical regulatory core for transdifferentiation. In fact, the concentration of 2‐oxoglutarate in cortical astrocytes was higher than that in striatal astrocytes (Figure 4B).

Among the genes highlighted in Figure 4A, *Gls* and *Glul* attracted our attention, because they regulate the balance between l‐glutamine and l‐glutamate, In addition, the expression of *Gls* is higher in striatal astrocytes, while that of *Glul* is higher in the cortical astrocytes (Figure 4C). We then tried to modulate transdifferentiation by overexpressing *Gls* with a retrovirus system or by inhibiting GLS activity with BPTES.^26^ As indicated in Figure 4D, overexpression of *Gls* inhibited the transdifferentiation of primary astrocytes from both the cortex and striatum, while BPTES facilitated transdifferentiation. The extracellular acidification rate (ECAR) and oxygen consumption rate (OCR) were then monitored with a Seahorse instrument to test this hypothesis. As predicted, overexpression of *Gls* inhibited OXPHOS and promoted glycolysis, while BPTES functioned oppositely (Figure 4E,F).

To improve the efficiency of transdifferentiation, we included 2‐DG and BPTES in 5C medium to form a new 7C medium. When the current 7C medium was used to replace 5C medium, ~50% more TuJ^+^ cells were obtained on day 12 during transdifferentiation (Figure 4G). When we infused 7C medium into the mouse cortex as described in Figure 1A, significantly more NeuN^+^Edu^+^ cells and GFAP^+^NeuN^+^Edu^+^ cells were observed around the injury sites (Figure 4H–J).

### 3.5 Biomaterials sustainably release key factors in 5C medium and induce transdifferentiation

**FIGURE 5:**
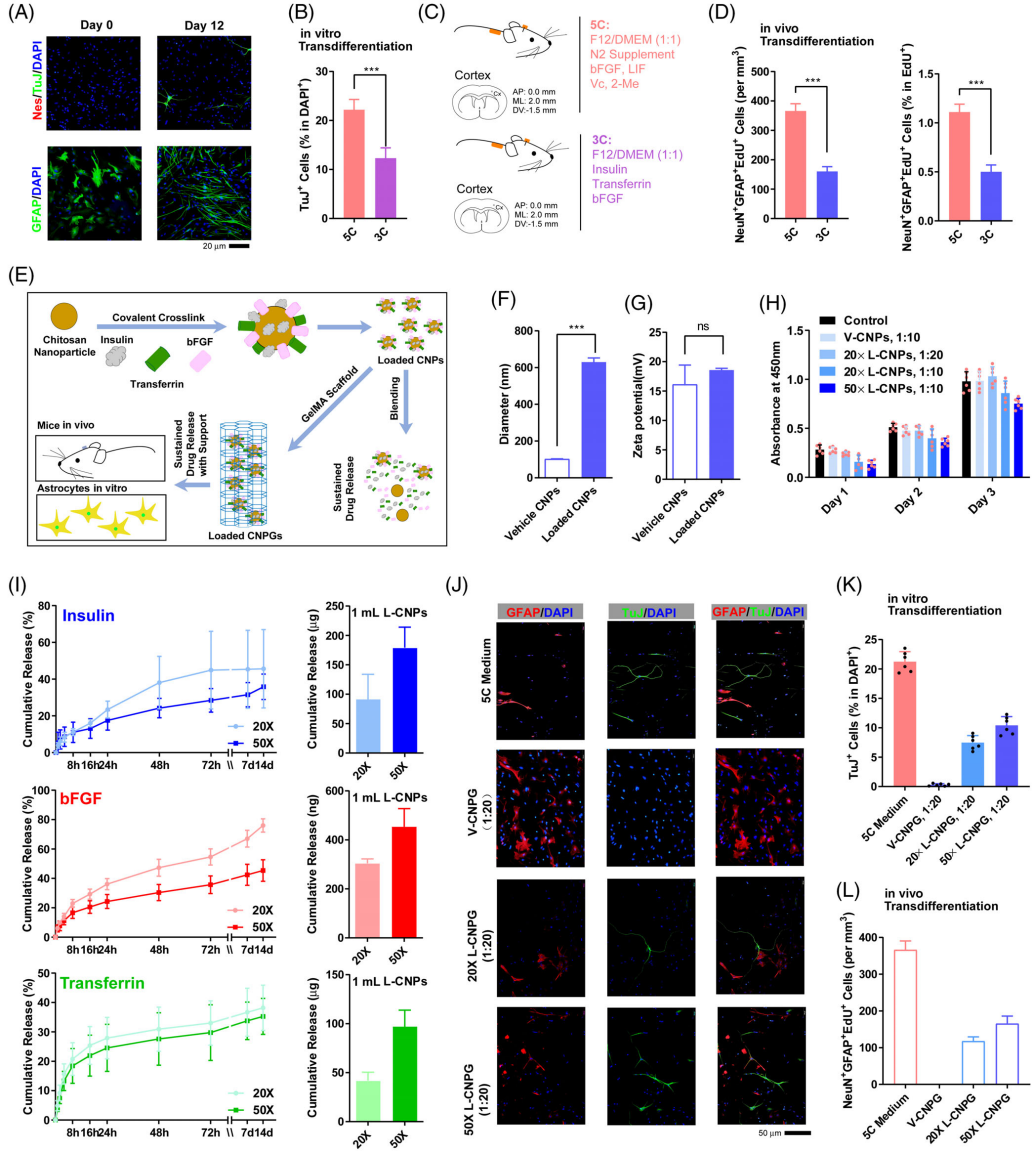
CNPG sustainably releases key factors in 5C medium and induces transdifferentiation in vitro and in vivo. (A,B) 3C medium‐induced transdifferentiation in vitro. (C,D) Schematic overview of 3C medium‐induced transdifferentiation in vivo (C). The amounts NeuN^+^GFAP^+^EdU^+^ cells were summarized in (D). (E) CNPs were loaded with insulin, bFGF and transferrin by covalent crosslinking. The vehicle or loaded CNPs were incorporated into GelMA scaffolds to generate vehicle and loaded CNPG, respectively. (F,G) The chemical and physical properties of the vehicle or loaded CNPs were characterized. (H) The cytocompatibility of the vehicle or loaded CNPs was evaluated with aCCK8 test. (I) Profile of insulin, bFGF and transferrin release from 1 mL loaded CNPs. (J,K) 5C medium and loaded CNPG induced transdifferentiation in vitro. Representative immunofluorescence images were provided in (J) and the results were summarized in (K). (L) 5C medium and loaded CNPG induced transdifferentiation in vivo. The amounts of NeuN^+^GFAP^+^EdU^+^ cells were summarized. Experiments were repeated for at least six times (*n* ≥ 6). Each animal group included at least six mice and at least seven frozen sections from each mouse (*n* ≥ 42) (L). Error bars represented standard deviations except standard errors in (D and M). Student two‐tailed *t*‐test was used. Additional statistic information could be found in Table S2.

3C medium which only included insulin, bFGF and transferrin in the basal DMEM/F12 (1:1) medium induced significant transdifferentiation both in vitro and in vivo, although the transdifferentiation efficiency was lower than that induced by 5C medium (Figure 5A–D).

To make the current system compactable to clinical application, we tried to use biomaterials, chitosan nanoparticles (CNP) and methacrylate gelatin (GelMA) hydrogels,^27^, ^28^, ^29^ to establish a sustainable drug release system, and deliver three key factors of 5C medium to the injury site (Figure 5E). Therefore, we loaded insulin, bFGF and transferrin onto CNP (loaded CNP, L‐CNP). The particle size and zeta‐potential of vehicle CNP (V‐CNP) were 103.933 ± 0.817 nm and 16.133 ± 2.694 mV (polydispersity index [PDI]: 0.233 ± 0.005), respectively, and those for L‐CNPs were 629.633 ± 18.579 nm and 18.567 ± 0.262 mV (PDI: 0.599 ± 0.022), respectively (Figure 5F,G). The PDI values near 0.2 represent a low tendency to aggregate and a narrow size distribution.^30^ The increased particle size and PDI value (decreased stability) proved that the drugs were loaded successfully. In addition, there was no significant cytotoxicity when different amounts of L‐CNP were used to achieve different final concentrations of the three growth factors (Figure 5H).

Both 20× and 50× L‐CNP possessed the abilities to release insulin, transferrin and bFGF for at least 14 days (Figure 5I). Although the cumulative release percentages of 50× L‐CNP were slightly lower than those of 20×, the total amount of released growth factors and the sustained release time of 50× were significantly better than those of 20× (Figure 5I).

Then the L‐CNP and V‐CNP were encapsulated into GelMA hydrogels to form loaded CNPG (L‐CNPG) and vehicle CNPG (V‐CNPG), respectively. CNPG was liquid before photo‐crosslinking and could be easily injected into the wound region. After exposure to 405 nm blue light for 2 min, the L‐CNPG were photocrosslinked and fit the shape of wounds which was beneficial to cell growth (Video S1).

The L‐CNPG and V‐CNPG were then used to induce transdifferentiation of primary cortical astrocytes. One hundred microlitres of CNPG (vehicle or loaded) was placed into the centre of a dish. After photocrosslinking, astrocytes were seeded. F12 medium was added every 2 days to maintain a volume of 1 mL (final concentration of 3C is 5‐fold or 2‐fold). As indicated in Figure 5J,K, ~20% of cells were TuJ^+^ on day 12 during 5C medium treatment, while ~10% of cells were TuJ^+^ when L‐CNPG were used. Similarly, L‐CNPG but not V‐CNPG induced significant transdifferentiation in the mouse cortex (Figure 5L).

### 3.6 Loaded CNPG facilitates the repair of brain injury in mice and pigs

**FIGURE 6:**
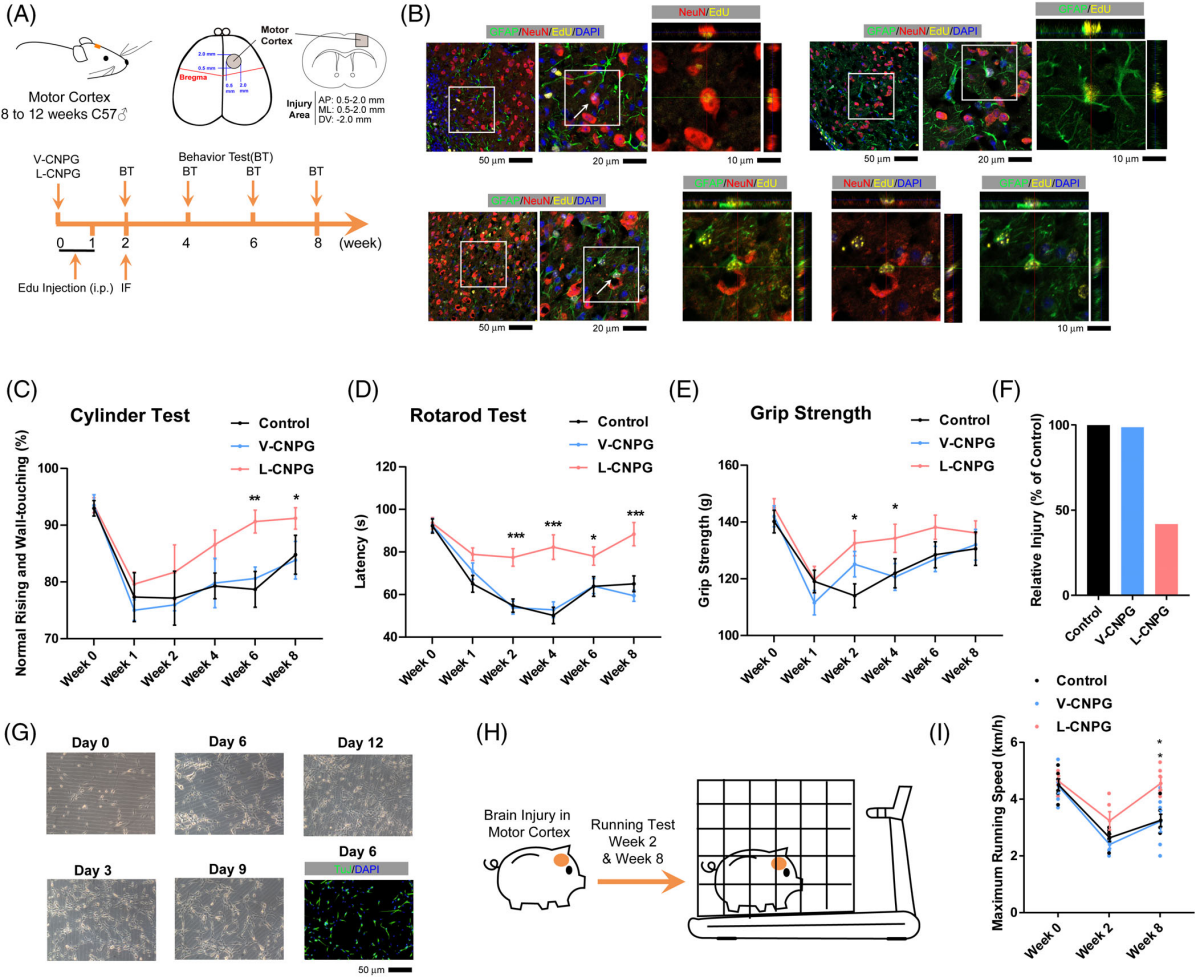
Loaded CNPG promoted motor function recovery after the brain injury. (A) Experimental design for mouse motor functional tests. A lesion cavity in the mouse right motor cortex was created by removing a block of cortical tissue (1.5 mm sphere) with an excavator spoon. The vehicle and loaded CNPGs were injected into the lesion cavity and photocrosslinked. Immunofluorescence was performed at week 2, and behavioural tests were further performed at weeks 2, 4, 6 and 8 to assess functional recovery. (B) Immunofluorescence for EdU (green), GFAP (yellow) and NeuN (red) in the cortex at 2 weeks post‐loaded CNPGs transplantation. There were a few NeuN^+^EdU^+^ cells, GFAP^+^EdU^+^ cells and NeuN^+^GFAP^+^EdU^+^ cells around the injury sites in the loaded CNPG group. (C–F) Cylinder (C), rotarod (D) and grip strength (E) tests were performed at week 2, 4, 6 and 8. Loaded CNPGs‐treated mice showed better recovery. The overall recovery was summarized in (F) after calculating the area between curves and the best performance (C–E). (G) 5C medium‐induced TuJ^+^ cells from primary astrocytes that were isolated from day 1 pigs. (H,I) A lesion cavity in the pig right motor cortex was created by removing a block of cortical tissue (7 mm sphere) with an excavator spoon. The vehicle and loaded CNPGs were injected into the lesion cavity and photocrosslinked. A treadmill test was performed at week 2 and 8 to assess functional recovery (H), and the results were summarized in (I). Each group included at least 12 mice (*n* ≥ 12) or at least six pigs (*n* ≥ 6). Error bars represented standard errors. Two‐way ANOVA was used. Additional statistic information could be found in Table S2.

With significant transdifferentiation in vivo and in vitro, we then investigated whether the L‐CNPG could rescue motor deficits caused by injury in the mouse motor cortex. A lesion cavity in the mouse right motor cortex was created. V‐ and L‐CNPG were injected into the lesion cavity, and photocrosslinked with 405 nm blue light for 2 min to fit the shape of the wound. To label newborn cells, mice were intraperitoneally injected with EdU once daily from the first to the seventh day after surgery (Figure 6A). Two weeks after surgery, immunofluorescence was performed, and we found that there were a few NeuN^+^EdU^+^ cells and NeuN^+^GFAP^+^EdU^+^ cells around the injury sites in the L‐CNPG group (Figure 6B).

The cylinder test, rotarod test and grip strength test were performed to evaluate locomotor asymmetry, motor coordination and function of the mouse forelimbs at different time points following surgery (Figure 6C–E). The lesion cavity in the mouse right motor cortex significantly impaired the performance of mice in these tests. The mice in the control and V‐CNPG groups gradually regained the locomotor symmetry and other abilities mentioned above, possibly due to self‐repair, but did not fully recover to the basal levels at the end of the current testing periods (week 8) (Figure 6C–E). In the L‐CNPG group, the facilitated recovery could be observed as early as week 2, and the performance of mice in these tests was close to basal levels. As summarized in Figure 6F, L‐CNPG reduced the injury by ~50%.

We then tried to examine whether the current L‐CNPG could also work in large animals, such as pigs. We first isolated primary pig astrocytes from the cortex of a 1‐day‐old pig, and 3C medium was used to treat the astrocytes for 2 weeks. TuJ^+^ cells were identified as early as on day 6 during the treatment (Figure 6G). A lesion cavity in the pig right motor cortex was created by removing a block of cortical tissue (7 mm sphere) with an excavator spoon. The V‐CNPG and L‐CNPG were injected into the lesion cavity and photocrosslinked with 405 nm blue light for 2 min to fit the shape of the wound and support cell growth. The pigs were placed on a treadmill with a stainless steel cage to restrain their movement (Figure 6H). The maximum running speeds were recorded when the pigs were forced to the end of the stainless steel cage by the treadmill. The pigs that received L‐CNPG performed better than those that received V‐CNPG (Figure 6I, Videos S2 and S3). Therefore, the current L‐CNPG facilitated the repair of brain injury in mice and pigs.

## 4 DISCUSSION

The transdifferentiation from astrocytes to neurons has recently been the subject of great debate. Two laboratories found that *Ptbp1* deletion converts glial cells to neurons in vivo,^31^, ^32^ which could not be reproduced by another two laboratories.^10^, ^23^ Yang's lab used CasRx to knockdown *Ptbp1* and converted Müller glia cells into retinal ganglion cells in mature mouse retinas.^31^ Fu's lab depleted *Ptbp1* in mouse astrocytes and successfully obtained neurons.^32^ The last two laboratories considered the previous reports as false‐positives that resulted from high titration of virus and virus leakage. An increasing number of groups have published their own opinions on the transdifferentiation from astrocytes to neurons.^11^ Although there is still no clear conclusion, the related laboratories and scientists agree that virus usage and lineage tracing are important for transdifferentiation.

To validate the transdifferentiation from astrocytes to neurons, lineage tracing transgenic mice that use the promoters of the marker genes of astrocytes to drive the expression of fluorescence protein or CreERT were used. However, some markers, such as GFAP which also serves as a marker for radial glial cells,^33^ are not specific enough. Some markers, such as *Aldh1l1* or *S100β* which label different sets of cells in the mouse brain,^34^ are restricted to particular subtypes of astrocytes. In addition, Chen's laboratory suggested that lineage‐tracing astrocytes are relatively difficult to convert, possibly because of previously experienced DNA cutting and annealing. Therefore, lineage tracing based on a single marker for astrocytes might not be the best choice to estimate the efficiency of transdifferentiation.

To determine the regional preference of the current transdifferentiation system, methods are required to quantify the transdifferentiation efficiency at least partially. In the current studies, GFAP and NeuN double‐positive cells (GFAP^+^NeuN^+^) were used to validate and semi‐quantify transdifferentiation because GFAP is general enough to label astrocytes, and no GFAP^+^NeuN^+^ cells can be identified in intact mouse brains or during the differentiation of NSCs or other progenitor cells. In addition, GFAP^+^NeuN^+^ cells were only identified in EdU^+^ cells, which is consistent with our previous report that cell proliferation is critical for transdifferentiation in vitro.^24^ The similar requirement for proliferation of transdifferentiation in vivo and in vitro suggested that the two types of transdifferentiation were in a similar cell fate conversion process. Since no cells other than astrocytes undergo transdifferentiation in vitro, transdifferentiation in vivo might not require other cells.

Cellular respiration is important in different processes of cell fate conversion including somatic cell reprogramming and neuronal transdifferentiation.^14^ The balance between l‐glutamine and l‐glutamate also influences the intracellular level of 2‐oxoglutarate,^35^ and 2‐oxoglutarate can maintain the pluripotency of embryonic stem cells by changing the transcriptional and epigenetic state of stem cells.^36^ We found that manipulating the expression of GLS can change the efficiency of transdifferentiation and cell metabolism, but how this happens requires further investigation.

DCX^+^ cells were used as proof of transdifferentiation in several studies on the transdifferentiation from glial cells to neurons.^4^, ^8^, ^37^, ^38^, ^39^ However, staining for DCX was not performed in some other studies.^7^, ^31^, ^32^, ^40^, ^41^ In a recent review,^42^ Chun‐Li Zhang's laboratory also summarized that DCX^+^ cells were not identified in many transdifferentiation studies. Gong Chen's laboratory usually detected DCX^+^ cells on day 5–7 after introducing NeuroD1.^4^ Since *Dcx* is a downstream target of NeuroD1^25^ and it is reasonable to detect DCX^+^ cells during NeuroD1‐induced transdifferentiation. However, upregulation of *NeuroD1* was not observed and overexpression of *NeuroD1* was not beneficial at least in our system and Gang Pei's system.^9^, ^24^ Thus, it is reasonable to suggest that DCX expression is not a must for transdifferentiation. BrdU and EdU labelling is a simple and broadly applicable gold standard to identify the newly generated neurons through transdifferentiation.^42^ The proliferation acceleration is required in the current transdifferentiation induced by 5C medium^24^ and we have used EdU to identify the intermediate state (GFAP^+^NeuN^+^EdU^+^) and the final state (NeuN^+^EdU^+^) of transdifferentiation.

## AUTHOR CONTRIBUTIONS

Conceptualization: Hui Zheng; Data Curation and Formal Analysis: Yuan Li, Tingting Yang, Yingying Cheng and Hao Sun, Funding Acquisition: Hui Zheng, Lin‐Ping Wu, Lin Guo, Lining Liang, Hao Sun and Changpeng Li; Investigation: Yuan Li, Tingting Yang, Yingying Cheng and Jinfei Hou did the majority of the experiments, all other authors except Hui Zheng also contributed to the investigation; Supervision: Hui Zheng; Writing – Original Draft: Yuan Li, Tingting Yang and Yingying Cheng; Writing – Review & Editing: Hui Zheng.

## ACKNOWLEDGEMENTS

This work was supported by the grants from the Science and Technology Program of Guangzhou (202007030003), the Strategic Priority Research Program of Chinese Academy of Sciences (XDA16010305), the Science and Technology Program of Guangzhou (202102020044 and 202102020183), the National Natural Science Foundation of China (32070717, 32170741, U21A20203, 32100472), the National Key R&D Program of China (2021YFA1100401 and 2021YFA1101304), the Science and Technology Planning Project of Guangdong Province (2020B1212060052) and the Research Funds from Health@InnoHK Program launched by Innovation Technology Commission of the Hong Kong SAR, P. R. China and Guangdong Pearl River Talents Program (2017GC010411).

## CONFLICT OF INTEREST STATEMENT

The authors declare that they have no conflict of interest.

## Supporting information


**Video S1.** Photocrosslinking for CNPG. Vehicle and loaded CNPs were mixed with GelMA hydrogels (4%) to generate vehicle and loaded CNPG, respectively. CNPG was liquid before photocrosslinking and could be easily injected into the wound. Exposure to 405 nm blue light for 2 min induced cross‐linking and curing of the biomaterials, which fit the shape of the wound well and supported cell growth.

**Video S2.** Representative treadmill test of one pig in V‐CNPG group. A suitable cage was placed onto the treadmill to keep the pig and make it run on the conveyer belt. The pigs were trained for three consecutive days with a fixed speed of 2.0 km/h in a period of 5 min on conveyer belt for five times. On day 4, pigs were tested with an accelerating speed from 0 to the maximum running speed that pigs can reach. The maximum running speed was recorded and analyzed. The pig received brain injury and V‐CNPG implantation, and video was recorded on week 8.

**Video S3.** Representative treadmill test of one pig in L‐CNPG group. A suitable cage was placed onto the treadmill to keep the pig and make it run on the conveyer belt. The pigs were trained for three consecutive days with a fixed speed of 2.0 km/h in a period of 5 min on conveyer belt for five times. On day 4, pigs were tested with an accelerating speed from 0 to the maximum running speed that pigs can reach. The maximum running speed was recorded and analysed. The pig received brain injury and V‐CNPG implantation, and video was recorded on week 8.

**Table S1.** Materials used in the current studies.

**Table S2.** Additional statistical information related to all figures.

**Data S1.** Supporting Information.

## Data Availability

A previously reported dataset (GSE184933) with scRNA‐seq data from different brain regions was used in the manuscript. All material requirements should be addressed to Hui Zheng (zheng_hui@gibh.ac.cn).
