# Supplementary material for: Low glutaminase and glycolysis correlate with a high transdifferentiation efficiency in mouse cortex
Source: Cell Prolif. 2023 Feb 14;56(5):e13422. doi: 10.1111/cpr.13422 (PMC10212695; doi:10.1111/cpr.13422)
Supplement: Supplementary file 6 — Data S1. Supporting Information. [file CPR-56-e13422-s006.docx]

**Supplementary Information**

**Materials and Methods**

**Particle size and zeta-potential**

The sizes and zeta-potential of particles were measured by the laser particle analyzer (Mastersizer 3000, England). After 10 μl sample was mixed with 1 ml deionized water, the diluted solution was loaded into the corresponding measuring container. Then the container was transferred into the particle analyzer and measured.

**CCK8 assay**

The cells were seeded into 96-well plate at a density of 1×10^3^ cells/well. Then the absorbance was measured at the different time points after treatment. For measurement, 10 μL CCK8 reagent (C0039, Beyotime Biotechnology) was added to each well. After incubation for 2 hours, the absorbance at 450nm was measured using microplate reader (GLPBIO, America).

**Release of growth factors from CNPs**

The drug-loaded nanoparticles were immersed in 2 ml PBS solution. After immersion, 1 ml solution was collected from tubes at different time points and then 1 ml PBS was added back into the tube after the collection. The collected solution was performed for measurement. The bFGF was detected by bFGF Elisa Kit (Thermo Fisher, No.EMFGF2), and the insulin and transferrin were detected by using the method of chemiluminescent immunoassay (Roche, Swiss).

**Isolation of primary astrocytes**

Primary astrocytes were generated from mice within 1 day after birth as previously described^2^. The mice were decapitated after disinfection. Their cerebra were placed into pre-cooled HBSS (Cytiva, SH30268.01) and cleaned for three times. Mouse brain was taken out from the cranium carefully under a stereomicroscope. Cortex and striatum were dissected from mouse brain under a stereomicroscope, then cut into small pieces and digested with 10 ml 0.15 % trypsin and 1 % DNaseI (Thermo Fisher,18047019) for 20 min at 37 °C. The digestion was stopped with 10 ml F12 medium containing 10 % fetal bovine serum and 1% penicillin/streptomycin (Sigma, V900929). The cells are filtered with 70 μm filter gauze and centrifuged at 300*g* for 5 min. The dissociated single cell was resuspended with astrocyte medium (Sciencell, AM-a1831), and a total of 1×10^6^ cells were plated onto a T75 culture flask. Forty minutes later, the supernatant was transferred into a new T75 culture flask for an additional seven-day culture. The flasks were then shaken at 260 rpm at 37°C for 12 hours, and the remaining astrocytes were digested from flasks and collected for the following experiments. All cells were tested for mycoplasma (Lonza, LT07-318), and were only used after obtaining negative results.

**Labeling with virus or EdU**

The virus solution was delivered into cortex through an infusion cannula at a flow rate of 40 nl/min by using the automated injection pump. A total volume of 1 μL of rAAV-CMV-EGFP-WPRE-pA (1× 1013 GC/ml, BrainVTA, China.) was injected into the cortex (AP: 0.0 mm, MP: 2.0 mm, DV: -1.5 mm). At the end of the injection, the pipette was held for another 10 min before slowly withdrawing to prevent any backflow. After virus injection for 14 days, 5C medium with EdU was infused into the cortex (AP: 0.0 mm, MP: 2.0 mm, DV: -1.5 mm) for 14 days. To label NSCs in the brain before surgery, EdU (5 mg/kg) dissolved in 0.9% sterile saline was injected intraperitoneally. EdU was administered to animals twice a day for four consecutive days.

**Cylinder test**

Mouse was placed into the transparent cylinder (10 cm diameter, 15 cm tall) on a transparent board. Rising and touching the sidewall with forelimbs were recorded with a camera from the top. The experiment was stopped after the mouse attempted to rise and touch the sidewall for over 30 times, which normally lasted for approximately 4-5 minutes. Analysis was conducted on 30 attempts of rising and touching. The normal rising and touching behavior were measured in terms of normal paw touches / total attempts × 100%.

**Rotarod test**

All mice were trained for 3 days and tested on the fourth day after habituation. On the first day, mice were trained with a fixed speed of 4 rpm in a period of 200s on the rotarod apparatus (Panlab LE8205) for 5 times. On day 2 to day 4, mice were trained and tested with an accelerating speed from 4 to 40 rpm during 120s for 5 times. The rods were cleaned with 70% ethanol after every trial to eliminate the olfactory cues. The time a mouse spent on the rod before falling off was recorded as latency and the mean value of 3 longest latencies was used for analysis.

**Grip strength test**

Grip strength was tested using an electronic grip strength meter (BIOSEB, BIO-GS3). After the forelimbs of mice gripped on the grid platform, with its torso in a horizontal position, their tails were grasped and pulled back steadily until the forepaws were released. The grip strength meter digitally displays the maximum force applied as the peak tension (in grams) once the grasp is released. Each trail was tested five times consecutively, with an interval of 20 seconds between each test. The mean value of 3 maximal readings was used for analysis.

**Treadmill test**

The pigs were placed on a treadmill in a closed cage to assess their running ability. A suitable cage was placed onto the treadmill to keep the pig and make it run on the conveyer belt. On the first day, pigs were trained with a fixed speed of 2.0 km/h in a period of five minutes on the conveyer belt for 5 times. On day 2 to day 4, pigs were tested with an accelerating speed from 0 to the maximum running speed that pigs can reach. The maximum running speeds were recorded for further analysis.

**Cellular energy metabolism**

The cellular energy metabolism was assessed using the Seahorse XF96 extracellular flux analyzer (Seahorse Bioscience, 101085-004) according to the instruction of instruments, with the simultaneous measurement of the OCR and the ECAR as the indicator of mitochondrial respiration and glycolytic conversion of glucose to lactate, respectively. For glycolytic conversion of glucose to lactate, the medium was replaced by XF assay medium without glucose or pyruvate. The assay workflow was as follows: 10 mM glucose was injected at 30 min, 1 μM oligomycin at 60 min, and 50 mM 2‐DG (a glucose analog) at 90 min. The following ECAR parameters were analyzed and calculated. An increase in ECAR after adding glucose indicated the level of glycolysis of the cells, while a decrease in OCR after adding oligomycin indicated the level of ATP production or OXPHOS of the cells.

**Immunofluorescence**

Cells or 10 μm frozen section were fixed with 4% PFA at room temperature for 15 mins and washed three times with PBS. After being blocked for 1 hour at room temperature, the primary antibodies were diluted in SignalUp™ Primary Antibody Dilution Buffer (Beyotime Biotechnology, P0262). After the incubation at 4°C overnight, samples were washed three times with PBS. The secondary antibodies were prepared with Secondary Antibody Dilution Buffer (Beyotime Biotechnology, P0265) and used at room temperature for 1 hour. Nuclei were stained with DAPI (Thermo Scientific, 62248). EdU staining was performed according to the instructions of Click-iT® EdU imaging kit (Invitrogen, C10340). Image collecting was conducted with Zeiss LSM 900.

**Statistical methods**

Experiments were repeated at least six independent biological repeats (n≥6). For *in vivo* transdifferentiation, each group included at least six mice and at least seven frozen sections from each mouse were analyzed (n≥42). In behavioral studies, each group included at least twelve mice (n≥12) or six pigs (n≥6). GraphPad Prism 7.0 was used to analyze and data compare, using Student’s t-tests, one-way ANOVA with Dunnett’s test as a post hoc test or two-way ANOVA with Bonferroni’s test as a post hoc test. Error bars represent standard deviations or standard errors, and “n” represents the number of independent experiments. “*”, “**”, and “***” denote significant differences (*P* < 0.05), (*P* < 0.01), and (*P* < 0.001) from the indicated control groups, respectively. All statistical information in the current studies was listed in Supplemental Table S2.
